# Common Salt in Intermittent Fever

**Published:** 1854-05

**Authors:** 


*Common Salt in Intermittent Fever.*

The use of common salt in ague has now for some time been advocated in Paris, especially by M. Piorry. It now appears, from a long report addressed to the Board of Trade of the French capital, by M. Willemin, late Sanitary Physician in the east, that the chloride of sodium is decidedly efficacious. The report concludes thus:

"1, Common salt has well marked febrifuge properties.

"2. In Damascus this salt stopped the fever six times out of seven cases; and even very small doses, as from two to four half-ounce doses in six ounces of water, were in most cases sufficient.

"3. This therapeutical agent is especially valuable in anæmic individuals, upon whom the marshy influence acts most severely; and the great cheapness of the salt should induce the profession to give their serious attention to its virtue in intermittent fever."

